# Horseback Journey

**Published:** 1878-02

**Authors:** 


HORSEBACK JOURNEY, every day, rain or shine, with some exhilerating object a-head, other than the mere healthfulness of it; in fact, no form of exercise can promise any marked good effect unless there is some other motive than that of health to give vivacity to the mind, and elasticity to the body, the spirits and the circulation.

